# Occurrence of bisphosphonate-associated osteonecrosis of the jaws in individuals with rheumatoid arthritis - a systematic review

**DOI:** 10.4317/medoral.26373

**Published:** 2023-12-27

**Authors:** Jefferson R Tenório, Daniella Estanho, Larissa Soares Lima da Silva, Andréa Vaz Braga Pintor, Marcela Baraúna Magno, Israel Leal Cavalcante, Bruno Augusto Benevenuto de Andrade, Lucianne Cople Maia

**Affiliations:** 1 Department of Oral Diagnosis and Pathology, School of dentistry, Universidade Federal do Rio de Janeiro; 2 Department of Pediatric Dentistry and Orthodontics, School of dentistry, Universidade Federal do Rio de Janeiro

## Abstract

**Background:**

To access the occurrence of bisphosphonate-associated osteonecrosis of the jaw (BAONJ) in individuals with rheumatoid arthritis (RA).

**Material and Methods:**

Observational studies that evaluated the occurrence of BAONJ in individuals with RA (BAONJ-RA) were considered for inclusion. Electronic searches were performed up to December 2022 in six databases and in the grey literature. The study selection, data extraction, and quality assessment of the included studies according to the Joanna Briggs Institute Critical Appraisal Checklists was performed. The certainty of evidence was evaluated using the GRADE approach.

**Results:**

Five studies were included three cohort and two cross-sectional. The sample size of subjects with RA ranged from 16 to 3201. Together, the studies presented 36 cases of BAONJ-RA. Prevalence of BAONJ-RA ranged from 0.094% to 56.25%. The incidence ranged from 0.4% to 2.21. Women between the 6th and 8th decade of life were the most affected. Alendronate (*n*=5) and zoledronic acid (*n*=9), orally and intravenously, respectively, were the most used bisphosphonates. The duration of bisphosphonates use ranged from 2.7 to 8 years. The certainty of evidence was very low.

**Conclusions:**

The occurrence of BAONJ-RA is low. However, the certainty of the evidence was very low for this outcome.

** Key words:**Bisphosphonate-associated osteonecrosis of the jaw, rheumatoid arthritis, diphosphonates.

## Introduction

Rheumatoid arthritis (RA) is a chronic autoimmune disease that affects the joints (1). RA has an approximate global incidence of 0.5% to 1% (1). RA has complex etiopathogenesis and environmental, genetic and epigenetic factors contribute to synovial inflammation, cartilage destruction and bone erosion (2). The joint destruction characteristic of RA leads to chronic pain, disability, musculoskeletal deficits, decline in physical function and quality of life, and increases the cumulative risk of comorbidities (1,2). In addition to joint involvement, extra-articular manifestations such as cardiovascular, pulmonary, gastrointestinal, renal and neurological involvement reflect the multisystemic nature of the disease (3).

Once RA is diagnosed, the overall goal of treatment is to achieve total remission or at least significantly reduce disease activity, preventing joint damage, disability, and extra-articular manifestations (2). Pharmacological management of RA includes several classes of drugs, such as antimalarials, disease-modifying antirheumatic drugs (DMARDs), nonsteroidal anti-inflammatory drugs (NSAIDs), tumor necrosis factor alpha (TNF-alpha) inhibitors, interleukin (IL) 6 inhibitors and glucocorticoids (1,2). The latter offer rapid symptomatic and disease-modifying effects, but are associated with serious long-term side effects, including osteopenia and osteoporosis (4). The American College of Rheumatology glucocorticoid-induced osteoporosis guidelines (2017) warn that patients with RA who use a dose greater than or equal to 2.5 mg of prednisone/day (or equivalent) for 03 months or more, are prone to have reduced bone mineral density and increased risk of fracture (5). Furthermore, alterations in calcium absorption and vitamin D metabolism that occur because of gastrointestinal and hepatic involvement also contribute to bone mineral impairment in these individuals (6).

In this context, bisphosphonates (BPs), drugs with antiresorptive properties, have been indicated for patients with RA who are at increased risk for fragility fractures (7). The use of these drugs has significantly reduced the incidence of vertebral and hip fractures (7). Nonetheless, an uncommon complication of the use of BPs is bisphosphonate-associated osteonecrosis of the jaws (BAONJ) (8). BAONJ is characterized by the presence of exposed bone, which is probed through an intraoral or extraoral fistula, for more than 8 weeks, in people with a history or current use of BPs (8). Individuals undergoing dentoalveolar surgeries are at increased risk for developing BAONJ (8).

Although it is a subject that has been extensively studied in oral medicine, to date, an evidence-based synthesis regarding the estimation of the global prevalence or incidence of BAONJ in individuals with RA, has not been reported yet. Thus, the aim of this study was to carry out a systematic review to answer the following focused question: What is the occurrence of bisphosphonate-associated osteonecrosis of the jaw in individuals with rheumatoid arthritis?

## Material and Methods

This systematic review is reported in accordance with the Preferred Reporting Items for Systematic Reviews and Meta-Analyses statement (http://prisma-statement.org.br) and registered on the PROSPERO database (CRD42022360369).

- Eligibility criteria

In this sense, studies in humans (P) with RA (E), and that developed BAONJ (O) were included. Thus, studies in humans with RA (or with other rheumatic diseases associated with the presence of RA, such as: Sjögren's syndrome, Caplan's syndrome, rheumatoid vasculitis, and Felty's syndrome) of any age, gender or ethnicity, who used BPs at any frequency, dose or route of administration were included. Observational studies with full-text available that reported the frequency of occurrence, prevalence, or incidence of BAONJ in patients with RA were also included. Conversely, studies that reported data on patients using RANKL inhibitors (E.g. denosumab), antiangiogenic medications (E.g. bevacizumab), TNF alpha inhibitors (E.g. etanercept, adalimumab, infliximab, certolizumab, golimumab) or with a history of radiotherapy in the head and neck were excluded. Literature reviews, case reports, case series, animal studies, laboratory studies, book chapters, letters to the editor and conference/congress abstracts were also excluded.

- Search strategy and information sources

Controlled vocabulary (MeSH terms) and free key words were used in the search strategies, which were defined based on aim of this review. In July 2023 a systematic literature search in PubMed, Scopus, Embase, Web of Science, Cochrane Library, and LILACS via Virtual Health Library electronic databases was performed. In addition, the grey literature was also searched through Google Scholar and OpenGrey. A monthly search alert was created to notify new studies according to the outlined search strategy up to February 2023. The search strategy was performed independently by 2 reviewers (J.R.T and L.S.L.S.) using the terms related to “bisphosphonate-associated osteonecrosis of the jaw”, and “rheumatoid arthritis” combined by the Boolean operators AND/OR, with no language or date restrictions (Table 1). A manual search on the included studies references lists was also performed to avoid missing any relevant publications. Similarly, experts in the field were contacted to retrieve possible relevant unpublished or ongoing studies data.

**Table 1:**
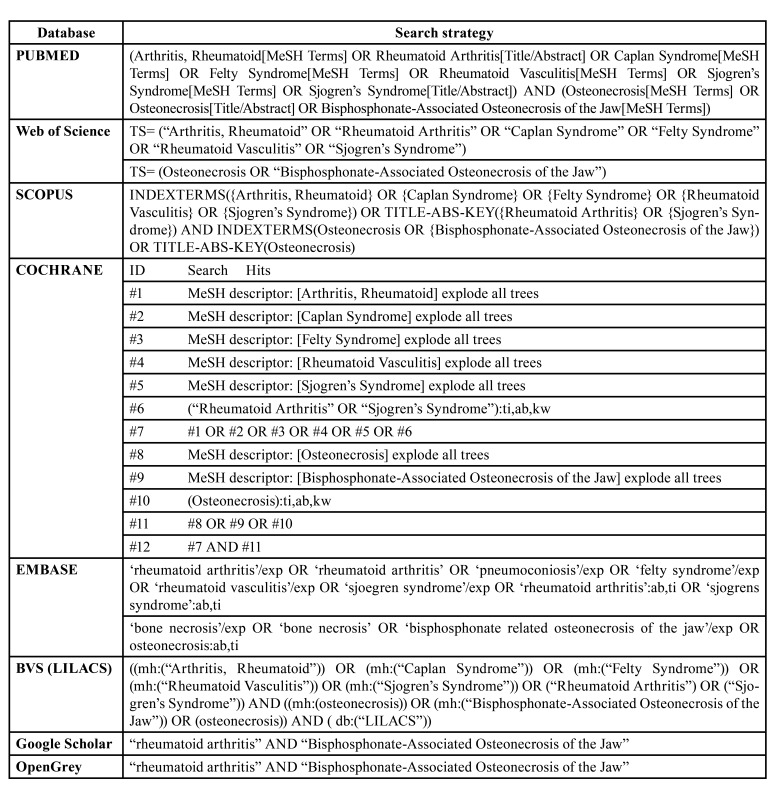
Search strategies.

- Study selection process

Articles identified in databases and by manual search were compiled into a bibliographic reference manager (Online version of EndNote, Version X7; Thomson Reuters, Philadelphia, PA). After automatic duplicated references removal, these records were exported to the Rayyan (https://www.rayyan.ai) software and underwent manual deletion of duplicates. Using Rayyan, two review authors (J.R.T. and L.S.L.S) performed the study selection, independently, through the evaluation of the titles and abstracts of all studies according to the eligibility criteria. Besides, when any title and abstract did not provide enough information for a definitive decision, the full text was retrieved and examined. Subsequently, all selected articles were read in full to confirm the eligibility. Any disagreements regarding the eligibility of studies for inclusion were resolved through consensus or with the help of a third author (L.C.M). Articles published in languages other than English and authors native idiom were translated using the Google® Translate tool (http://translate.google.com.br).

- Data collection process

The following data were collected: author, year, country, type of study, total sample size (n), RA sample size, number of cases of BAONJ in individuals with RA (BAONJ-RA), prevalence/incidence of BAONJ (according to the type of study), participants age range and sex, type of BP used, route of administration (oral/intravenous), duration of use, and history of previous oral surgical procedure (when available). When data were not fully available, participants in this systematic review contacted the Correspondence of the article through weekly emails for four consecutive weeks.

- Study risk of bias and certainty of evidence assessment

The risk of bias in the included studies was assessed independently by two reviewers (D.E. and L.S.L.S) using the Joanna Briggs Institute (JBI) Critical Appraisal Checklists for Observational Studies (Cross-sectional and Cohort) (9). For cross-sectional studies, all 10 items were applied. For cohort studies, 10/11 questions were considered applicable. Item 10 of the JBI for cohort studies (related to strategies to deal with incomplete follow-up) was considered not applicable, since the data needed for the study do not require the evaluation of cases with incomplete follow-up. Disagreements between the reviewers about the quality assessment were solved by a third author (A.V.B.P.).

The certainty of evidence was evaluated using the GRADEpro tool (10). Risk of bias, inconsistency, indirectness, imprecision, suspicion of publication bias, presence of large effect, plausible confounders and dose response gradient were the items considered to rate the overall certainty of evidence (11,12). All the judgments were adapted to qualify the evidence synthesized in a narrative way (13).

- Synthesis methods

Initially, the characteristics of the included studies were summarized and tabulated using Excel spreadsheets (Excel®, Microsoft, USA). Studies were grouped for synthesis based on study design (cross-sectional or cohort), incidence/prevalence, route of administration (oral/intravenous) and duration of use (in years) of BPs.

## Results

- Study selection

**Figure 1:**
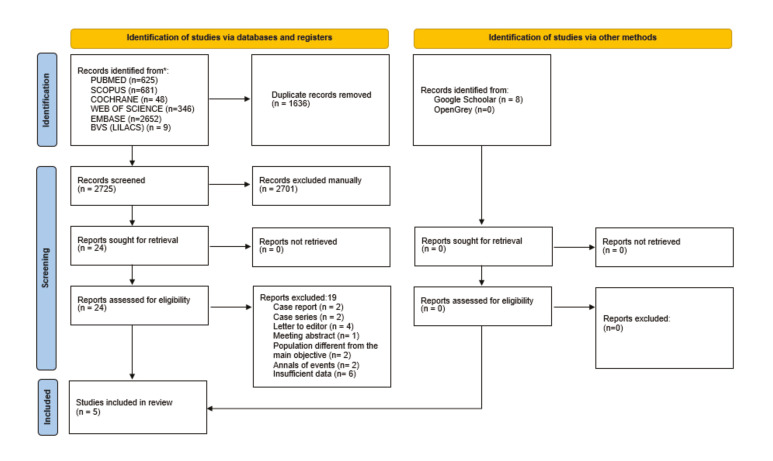
PRISMA flowchart.

The flowchart of the search selection procedures is shown in Fig. 1. Of the 4.361 articles retrieved through database searches, 1.636 duplicates were removed. A total of 2.701 records were excluded by reading titles and abstracts, remaining 24 articles that were sought for retrieval. Nineteen reports (Supplement1) were excluded after full-text reading and 05 studies were finally included (14-18). Eight studies were identified via grey literature, but none met the eligibility criteria by reading titles and abstracts.

- Studies characteristics

Two cross-sectional studies (15,17) and 03 cohort studies (14,16,18) published between 2014 and 2021 were included in this systematic review. The studies were carried out in Taiwan (14,16), Japan (15), Spain (17) and South Korea (18) and a total of 5009 patients with RA were analyzed. The sample size of subjects with RA in the cross-sectional studies ranged from 16 (17) to 1236 (15) patients, and in cohort studies ranged from 226 to 3201 patients. The incidence of BAONJ-RA was low, ranging from 0.4% (18) to 2.21% (14). Prevalence ranged from 0.094% (15) to 56.25% (17). Age ranges in gross values for individuals with BAONJ-RA, when available, ranged from 65 (15) to 91.51 (16) years. The female gender was predominant in all studies for which this data was available (15,16,17). Alendronate (*n*=5) (15,16) and zoledronic acid (*n*=9) (17), orally and intravenously, respectively, were the most used BPs by the individuals with BAONJ-RA. In cross-sectional studies (15,17) the minimum time of use of BPs was 5 years. Only one cohort study (16) reported the duration of use of such drugs by the patients with BAONJ-RA, which ranged from 2.76 to 7.01 years. This same study showed that there was a higher proportion of occurrence of ONJ among individuals taking alendronate who had a history of tooth extractions in the year prior to the final date of data collection, with a crude odds ratio of 10.46. A cross-sectional study (17) showed that 10/16 patients with RA had a history of tooth extractions, of which 09 developed BAONJ. The detailed characteristics of the studies are presented in Table 2.

- Risk of bias/quality of studies

The cohort studies (14,16,18) showed clarity and methodological rigor, especially in terms of similarity and patient recruitment, exposure measures, identification of confounding factors, absence of the outcome at baseline, outcome measures, follow-up time and statistical analysis. However, for two of them (14,16) it does not seem clear whether there were strategies to deal with the confounding factors (presence of diabetes mellitus, smoking, among others) and the loss of some follow-ups.

The two cross-sectional studies apparently had problems with outcome measures, in one of the studies (15), the diagnosis of osteonecrosis of the jaws was conditioned to the answer to a questionnaire answered by the patient, and in the other, the diagnostic criteria employed were not detailed (17). The latter (17) also had some methodological flaws, including problems with inclusion criteria and sample characterization, exposure measures, strategies to deal with confounding factors, and statistical analysis. JBI’s critical appraisal results according to the type of each study are listed in Supplement 2 and Supplement 3.

**Table 2:**
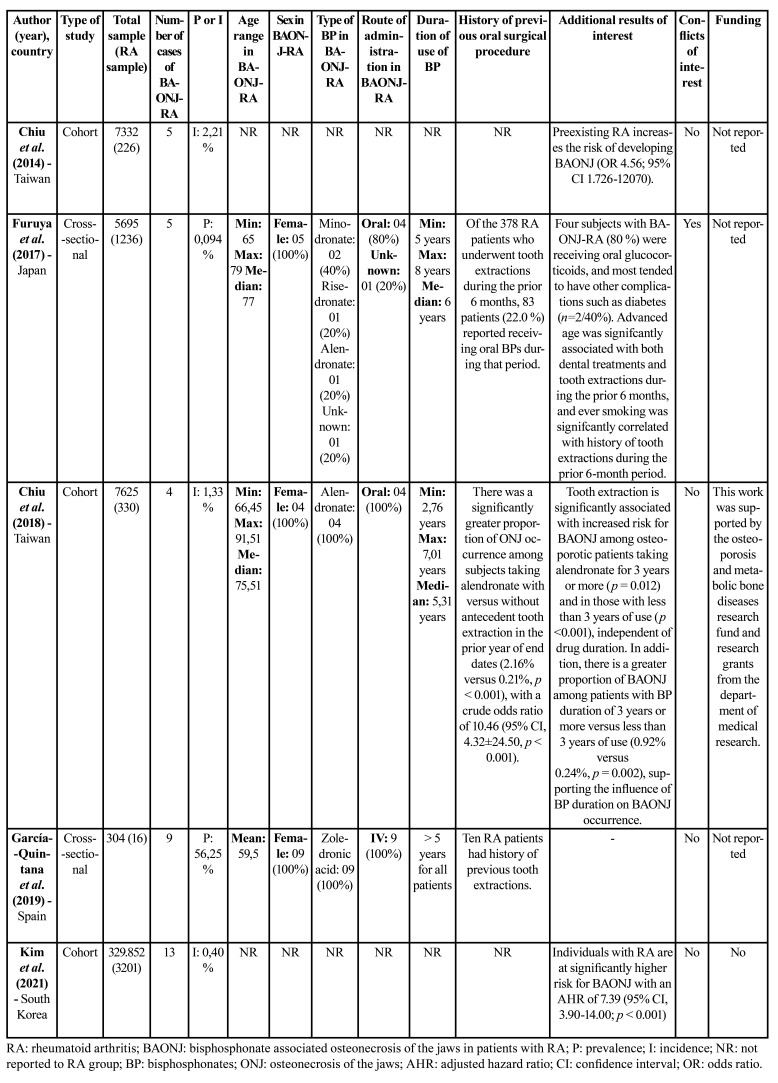
Characteristics of the included studies.

- Results of the studies

A narrative synthesis of the included studies was performed and the results of the individual studies are shown in Table 2.

Only one cohort study presented the variables studied for the group of individuals with RA who developed BAONJ (BAONJ-RA). However, in general, the incidence of BAONJ-RA was low, reaching a maximum of 2.21% (*n*=5), in a study (14) with a sample of 226 individuals with RA. Chiu *et al*. (2014) (14) showed that having RA increased the risk of developing BAONJ by 4.56 times. In the study by Kim *et al*. (2021) (18), this estimated risk was 7.39.

Furuya *et al*. (2018) (15) evaluated 1236 patients with AR, of which 5 developed BAONJ, resulting in a reduced prevalence of this condition. On the other hand, a cross-sectional (17) study showed a marked prevalence of BAONJ-RA, reaching 56.25%.

- Certainty of evidence

The certainty of evidence was rated as very low (Table 3). In cross-sectional studies some limitations related to imprecision (number of events - BAONJ cases), indirectness (outcomes were not similarly defined in these studies), which also reduced the certainty of evidence in terms of inconsistency. The included cross-sectional and cohort studies had methodological flaws that could alter the results, reducing the evidence due to the risk of bias.

**Table 3:**
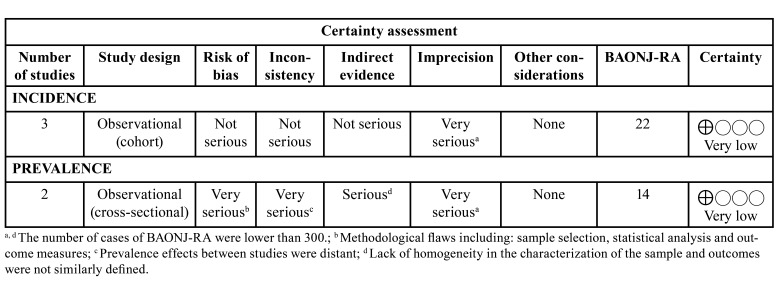
Certainty of evidence.

## Discussion

BAONJ is a bone disease that is continuously studied in oral medicine and, although uncommon, it can present with chronic exposure of necrotic bone in the oral cavity, pain, fistula, oroantral communication and pathological fracture (8). The occurrence of this condition within the context of the RA patient, who already has chronic joint pain, morning stiffness and a propensity for disability and deformity, may further reduce the quality of life of affected individuals (1,2). The focused question of this systematic review was aimed at clarifying the global prevalence and incidence of BAONJ in individuals with RA. The present review synthesized the evidence on this issue and found that most of the included studies have shown low prevalence and incidence of BAONJ in individuals with RA (14,15,16,18). Although the study by García-Quintana *et al* (2019) showed a discrepant prevalence (56.25%), this data needs to be carefully analyzed, since its methodology included a group of patients with osteonecrosis of the jaw (ONJ) (*n*=24) and another without this condition (*n*=280); within the sample of patients with ONJ, a small portion had RA as the main comorbidity (*n*=9).

The relationship between RA and BAONJ has already been shown in animal models and in human studies (19,20). Although it seems to be an uncommon condition, the dentist must be aware that individuals with RA can experience worse oral health conditions due to factors related to the underlying disease (21) and, in addition, present foci of oral infection that act as a trigger for the development of BAONJ in patients using BP.

In general, the included studies showed some methodological limitations, many of them related to the nature of observational studies. However, given the multifactorial characteristic of BAONJ, all studies (14-18) pointed to confounding factors that may influence the development of BAONJ, including: diabetes mellitus and smoking. However, two studies (14,16) did not show strategies to deal with the presence of these factors. It is possible that these conditions influenced the results found.

The demographic profile of patients with BAONJ-RA consisted predominantly of women between the 6th and 8th decade of life. This was an expected finding, since both RA and osteoporosis are more common in postmenopausal women (22). However, this data deserves to be carefully analyzed, given the reduced number of events found (i.e. number of cases of BAONJ).

Dentoalveolar surgeries are the main local factors associated with the development of BAONJ (8). Unfortunately, most of the studies included were not clear about the prior performance of oral procedures specifically in individuals with RA using BPs. Furthermore, other local factors that are commonly associated with an increased risk for developing BAONJ (anatomical factors and concomitant oral disease) (8) were also not addressed.

Another issue that needs to be mentioned is the quality of the outcome measures, that is, how the diagnosis of BAONJ was performed. The cross-sectional studies included showed a lack of clarity in this regard (15,17). It is important to emphasize that the diagnosis of BAONJ is based on well-established clinical criteria and that radiographic imaging is an auxiliary resource, especially in more advanced cases, to define the extent of bone involvement (8).

In this sense, given the low level of evidence, the authors suggest that studies with the following criteria can be carried out: 1) specifically select samples from patients with RA as the main systemic disease; 2) clearly show the diagnostic criteria for BAONJ; 3) describe local risk factors for BAONJ; 4) report in detail the type of BPs used, as well as dose, route of administration and duration of use; 4) describe in detail the confounding factors and show robust strategies to deal with them.

It's important to note that certain antirheumatic medications, such as TNF-alpha inhibitors, have also been associated with osteonecrosis of the jaw (23). There is also a potential risk of osteonecrosis of the jaw with other antiresorptive and antiangiogenic medications. This led the American Association of Oral and Maxillofacial Surgeons to define the term 'medication-associated osteonecrosis of the jaw’ (8). Therefore, in this study, we specifically used the term BAONJ to focus exclusively on patients using BPs. Additionally, it's worth mentioning that the study sample likely included individuals who initially appeared to be sTable but may have experienced relapses, necessitating the use of steroids for an extended period, warranting additional bone protection therapy. For those individuals who do not respond to first-line treatment, biologic medications may become a necessary option.

This systematic review has some limitations that need to be addressed, among them is the lack of some data specifically related to individuals with RA. Furthermore, the five studies (14-18) included were methodological heterogeneous in some aspects, such as: study design, type and route of administration of BPs used. Thus, it was not possible to carry out a quantitative synthesis through meta-analysis.

## Conclusions

In summary, the occurrence of BAONJ in individuals with RA is low. However, this data needs to be analyzed carefully, since the certainty of the evidence was very low for this outcome. We recommend conducting studies with high methodological quality to improve the evidence.
